# Comment on “Myosteatosis and Muscle Loss Impact Liver Transplant Outcomes in Male Patients With Hepatocellular Carcinoma” by Lu et al.—The Authors' Reply

**DOI:** 10.1002/jcsm.13762

**Published:** 2025-03-05

**Authors:** Di Lu, Zhihang Hu, Hao Chen, Xiao Xu

**Affiliations:** ^1^ Department of Hepatobiliary & Pancreatic Surgery and Minimally Invasive Surgery Zhejiang Provincial People's Hospital, Affiliated People's Hospital of Hangzhou Medical College Hangzhou China; ^2^ NHC Key Laboratory of Combined Multi‐Organ Transplantation Hangzhou China; ^3^ Zhejiang University School of Medicine Hangzhou China; ^4^ Institute of Translational Medicine Zhejiang University Hangzhou China

To the Editor

We appreciate the interest and insightful comments by Kamiliou et al. [1] regarding our recently published study [2]. Our work incorporated 756 participants from 3 transplant centres and identified that pre‐transplant myosteatosis aggravated the adverse impact of sarcopenia on liver transplant outcomes in male patients with hepatocellular carcinoma (HCC). In terms of the prevalence of myosteatosis, they suggested that the low prevalence we reported could be attributed to the inclusion of Asian patients with chronic hepatitis B‐related HCC. After reviewing their work [3], we agree that the variance in the prevalence of myosteatosis is worth exploring. Also, the variance in myosteatosis prevalence may be influenced by assessment criteria. By using skeletal muscle radiodensity (SMRA) [2, 4] or intramuscular adipose tissue content (IMAC) [5, 6], studies usually reported substantially different positiveness rates. Regarding the different outcomes by gender, our study focused on the prognostic value of skeletal muscle parameters primarily in a male cohort. Yet, it is still insufficient to conclude that myosteatosis is unrelated to post‐liver transplantation outcomes in females. Future research should address this question in relatively larger female cohorts, particularly in HCC where male patients take up the majority. Furthermore, they reported a higher incidence of hepatic encephalopathy in patients with myosteatosis [7], which is supported by other studies [8, 9]. Given that our study focused on the association between skeletal muscle and post‐transplant outcomes, we did not adequately evaluate the association between myosteatosis and hepatic encephalopathy. Nonetheless, we appreciate their efforts on this important issue as hepatic encephalopathy remains a major concern in patients with end‐stage liver disease. About diabetes, we observed a higher prevalence of myosteatosis in the diabetic population compared to the non‐diabetic group (34% vs. 26%). The mean SMRA was significantly lower in the diabetic group than in non‐diabetic patients (39.8 HU vs. 41.0 HU, *p* = 0.047), partially confirming an association between diabetes and myosteatosis.

In this latest work, we established a streamlined approach applicable to both sarcopenic and non‐sarcopenic recipients, with the aim of stratifying recipients based on distinct prognostic risks to guide clinical diagnosis and treatment. Of note, our team previously demonstrated that both myosteatosis and sarcopenia independently and additively increase mortality risk among recipients of split liver transplantation [10], complementing the conclusions of this study. While our study assessed peri‐transplant muscle mass changes and their impact on prognosis in non‐sarcopenic patients, we found no significant effect of postoperative myosteatosis. Interestingly, our results also indicated that preoperative myosteatosis was not associated with the rate of postoperative muscle loss. This distinction underscored a fundamental difference between sarcopenia and myosteatosis, suggesting that these conditions may impact clinical outcomes through distinct mechanisms. Given the authors' keen interest in the prevalence and diagnosis of myosteatosis, we look forward to future collaborations aimed not only at standardizing the diagnostic criteria and assessment procedures for myosteatosis but also at achieving precise, individualized diagnoses that account for race, sex, age and comorbid conditions.

## Conflicts of Interest

The authors declare no conflicts of interest.
